# The Neuropsychological Profile of Severe and Enduring Anorexia Nervosa: A Systematic Review

**DOI:** 10.3389/fpsyg.2021.708536

**Published:** 2021-08-02

**Authors:** Catherine Broomfield, Kristin Stedal, Stephen Touyz

**Affiliations:** ^1^The School of Psychology, University of Sydney, Sydney, NSW, Australia; ^2^Regional Department for Eating Disorders, Oslo University Hospital, Ullevål, Norway; ^3^Inside Out Institute, Charles Perkins Centre, The University of Sydney, Sydney, NSW, Australia

**Keywords:** anorexia nervosa, neuropsychology, severe, enduring, chronic

## Abstract

Characteristics of Severe and Enduring Anorexia Nervosa (SE-AN) are being investigated to differentiate the patients experiencing SE-AN from those at earlier stages of the AN disease. The current systematic review was the first step in exploring neuropsychological functioning as a potentially identifying characteristic for long-term presentations. With a subgroup of AN patients reflecting a unique neuropsychological profile that is proportionate to the quantity of patients that go on to develop SE-AN, it was the aim of this review to assess neuropsychological functioning in the later stage of the disease. In accordance with PRISMA guidelines, a literature search was conducted using four electronic databases (PsycINFO, MEDLINE, Web of Science, and Scopus) for neuropsychological research on AN participants with a seven or more year illness duration. Datasets that met inclusion criteria were screened for SE-AN participants (*N* = 166) and neuropsychological data extracted together with potentially confounding variables and information required to conduct a quality assessment. In research investigating decision-making, participants with a SE-AN presentation demonstrated significantly lower functioning compared to healthy controls. There was conflicting evidence for differences in intellectual functioning and set-shifting abilities with no variability indicated in central coherence, memory, attention, reasoning, or processing speed. If findings from this preliminary analysis are confirmed through empirical research, implications include earlier identification of SE-AN patients and more effective treatment development.

## Introduction

Anorexia nervosa (AN) is a complex illness that is characterised by a restriction in nutritional intake relative to the individual. With both physical and mental manifestations, notable features of the illness include an intense fear of becoming fat and a low body weight (American Psychiatric Association, 2013). Additionally, there is often a distortion to the way the individual judges his or her own body with little evidence of bias in the perception of body representation more generally (Mölbert et al., 2018; Behrens et al., 2020). What further complicates the illness is the variability in its presentation. Despite two-thirds of females with AN expected to recover within 22 years of onset (Eddy et al., 2017), ~20% of individuals with the illness experience an enduring disorder (Steinhausen, 2002). As a result of these different outcomes, efforts have been made to distinguish the various subgroups within AN in order to stage the illness based on some form of indicator(s) (Maguire et al., 2008; Treasure et al., 2015). The subgroup that has been deemed the most challenging to treat with the most prolonged trajectory is Severe and Enduring AN (SE-AN; Touyz et al., 2013; Ciao et al., 2016). The implications that stem from experiencing this presentation has been explored in a recent review by Wonderlich et al. (2020).

With an increased interest in the severe and enduring subgroup, there is a debate with respect to how individuals should be labelled and identified (see Broomfield et al., 2017 for a review). Given the uncertainty, numerous aspects of SE-AN are being investigated so that features of the disease can be better understood. When considering research into characterising SE-AN, Wildes et al. (2017) outlined the importance of taking into account multiple facets of severity and chronicity, which would be beneficial to both research and clinical practise. Presently, the most common criterion for identifying patients with SE-AN is illness duration, with a 7-year period being the most frequently used in the literature (Broomfield et al., 2017). Through discovering alternative indicators of SE-AN to illness duration, additional research has the potential to help navigate more effective treatment options that target these presentations and provide enhanced outcomes for the individuals who experience this illness. Clinical features, including quality of life, has been explored in research (see Mitchison et al., 2013; Bamford et al., 2014; Le Grange et al., 2014) with this outcome being a focus in new models of treatment for SE-AN (see Touyz et al., 2013; Touyz and Hay, 2015). Additionally, preliminary research into the social and emotional functioning of these patients has begun to emerge (see Arkell and Robinson, 2008; Franko et al., 2016; Utzinger et al., 2016). However, what is crucial to understand is the neuropsychological impact of this long-term presentation, given duration of illness in other mental health conditions has been associated with a decline in cognitive functioning (Melloni et al., 2019; Galimberti et al., 2020; Liu et al., 2021).

In the cognitive-interpersonal maintenance model of AN [originally proposed by Schmidt and Treasure (2006) and then updated by Treasure et al. (2020)], premorbid vulnerabilities such as obsessive compulsive traits are described as contributing to the development and maintenance of AN. There was evidence to support this model by a study suggesting that obsessive-compulsive disorder that was premorbid to the onset of AN symptoms was associated with a poorer outcome in adolescent patients (Carrot et al., 2017). It has been proposed that what underpins the obsessive-compulsive traits found in AN patients is atypical cognitive functioning, or more specifically, a rigid cognitive processing style (Schmidt and Treasure, 2006; Treasure and Schmidt, 2013). If this theory is valid and a more rigid cognitive processing style is what plays a role in developing and maintaining the disease, it can be presumed that this cognitive style will be particularly salient in individuals with a severe and enduring presentation given the longevity of the illness. Logically, this theory needs to be tested with reference to individuals with SE-AN given this may provide additional evidence as to what cognitive style is most vulnerable to the maintenance of AN.

The two cognitive processes found to be particularly affected and consistently reported as impaired in patients with AN are cognitive flexibility and a bias toward processing local as opposed to global information (Tenconi et al., 2010; Shott et al., 2012; Kanakam and Treasure, 2013; Treasure and Schmidt, 2013). Cognitive flexibility, or what is more commonly referred to as set-shifting, is the ability to effectively move back and forth between different mental tasks (Wu et al., 2014). This requires the individual to inhibit previous behavioural response patterns in order to comply with changing task parameters to achieve a desired outcome (Ely et al., 2016). In a real-world context, patients with AN tend to adhere to strict and rigid practises in order to avoid gaining weight despite being severely underweight and undernourished. It has been identified through numerous studies that patients with AN experience difficulties adapting to new rules in a changing environment (see Roberts et al., 2007; Tchanturia et al., 2011; Talbot et al., 2015). The second process found to be most prominently affected by the AN illness is a cognitive bias in information processing that relates to the concept of central coherence. It has been hypothesised that individuals with AN experience a weak central coherence, which relates to paying excessive attention to details as opposed to the bigger picture (Lopez et al., 2008; Brown et al., 2018). This is in contrast to healthy individuals who tend to show a bias toward more global features at the expense of specific features (see Navon, 1977; Weinbach et al., 2017; Hamatani et al., 2018).

Evidence relating to changes in both set-shifting and central coherence has been assessed in the generic condition of AN (see Tenconi et al., 2010; van Autreve et al., 2013; Roberts et al., 2016) and in comparison to individuals who have recovered from the illness (see Tchanturia et al., 2002; Bosanac et al., 2007; Danner et al., 2012). One theory that has been proposed by Ely et al. (2016) is that cognitive deficits may only exist in certain subgroups of AN, which was suggested through the discussion of research findings into cognitive inhibition in patients with remitted AN. In research using a cluster analysis approach, Rose et al. (2016) found that adolescent patients with AN showed variability in their neuropsychological profile with a unique cluster demonstrating low average to average performance across 15 neuropsychological measures. Specific deficits were particularly evident on measures capturing cognitive flexibility, central coherence and verbal abilities compared to healthy controls. Of note is that 19% of the AN sample exhibited this unique neuropsychological profile (Rose et al., 2016). When considering the neuropsychological functioning of adults, a study by Renwick et al. (2015) found three distinct clusters of patients with AN. Each cluster varied in their neuropsychological abilities except for central coherence, which was found to be consistently weak across all AN patients. One cluster, which consisted of 17% of participants in the study, was found to have an overall poor performance compared to the other two clusters, particularly in relation to measures of executive functioning (Renwick et al., 2015). The possibility of only distinct subgroups of AN patients exhibiting neuropsychological weaknesses has also been suggested by Overas et al. (2017), whilst discussing the research findings of Renwick et al. (2015).

To the authors' knowledge, no research outside of case reports has exclusively focused on the neuropsychological functioning of patients with an AN illness duration of 7 or more years. However, delving deeper into the two clusters found in the studies of Rose et al. (2016) and Renwick et al. (2015), there may be some similarities between these participants despite one study investigating functioning in adolescents and the other in adults. The 19% (Rose et al., 2016) and 17% (Renwick et al., 2015) of AN participants consisting within these clusters may represent the 20% of patients that go on to develop a life-long battle with the illness (Steinhausen, 2002). It is therefore a possibility that these specific participants demonstrate a characteristic of SE-AN i.e., a potentially unique neuropsychological profile. This hypothesis seems particularly plausible in the context of research suggesting that a longer duration of illness may be associated with certain neuropsychological deficits compared to individuals with shorter durations of the illness (see Abbate-Daga et al., 2011; Lang et al., 2014; Treasure et al., 2015). Additionally, a recent hypothesis proposed by van Elburg et al. (2021) suggested that poor decision-making skills underlie, fuel, or contribute to the mental capacity of patients with SE-AN, which may influence their ability to recover. With no empirical evidence to support or refute this theory, it was crucial to explore this possibility in the current study.

When integrating the neuropsychological research to date, the rationale for the current study becomes threefold. First, research conducted on adults appears to have been met with mixed findings with some evidence of superior (Lopez et al., 2010), inferior (Adoue et al., 2014; Blumberg et al., 2014; Chan et al., 2014), or relatively indifferent (Overas et al., 2017) cognitive functioning in patients with AN compared to healthy controls. Examining neuropsychological abilities in different subpopulations of AN may provide a more clear understanding as to how the disease impacts the functioning of the brain and which patients are more susceptible to these neurocognitive effects. Second, a longer duration of illness has been suggested as a variable that may be responsible for neuropsychological impairment (Saure et al., 2020) with this hypothesis supported by findings in a recently conducted meta-analysis by Stedal et al. (2021). Third, along with neuropsychological variability determined in a cluster of adolescents with AN (Rose et al., 2016), cluster analysis on an adult population has also indicated neuropsychological variability in a specific subgroup of patients (Renwick et al., 2015). In the current line of research, it is hypothesised that the unique neuropsychological profiles of these participants represent a characteristic of SE-AN given the fairly symmetrical proportion of these clusters relative to the proportion of individuals that experience a long-term trajectory of AN.

It was therefore the aim of the present systematic review to synthesise the available research on the neuropsychological performance of adults experiencing SE-AN. Despite research to date suggesting set-shifting and central coherence to be the most significantly impaired neuropsychological abilities observed in patients with AN, the current analysis canvassed all areas of functioning to provide a broad understanding of the impact of the severe and enduring presentation. Based on previous meta-analytic investigations by Lang et al. (2014) and research findings by Talbot et al. (2015), set-shifting has been found to be particularly affected by duration of illness. It was therefore predicted that the current analysis would find set-shifting impaired in participants with SE-AN. No other hypothesis could be devised for other abilities given there is virtually no neuropsychological research currently dedicated to SE-AN.

## Methods

### Search Strategy

This systematic review was conducted in accordance with PRISMA guidelines (Moher et al., 2009), which provides a methodological framework and protocol for the research. A comprehensive and exhaustive search was conducted using four electronic databases (PsychINFO, Medline, Web of Science, and Scopus) in order to identify research relating to the neuropsychological functioning of individuals with SE-AN. All dates, settings and designs of peer-reviewed journal articles were included in the search with the exception of reviews. The original search was conducted on 19 August 2020 with an updated search conducted on 16 April 2021.

**Figure 1:**
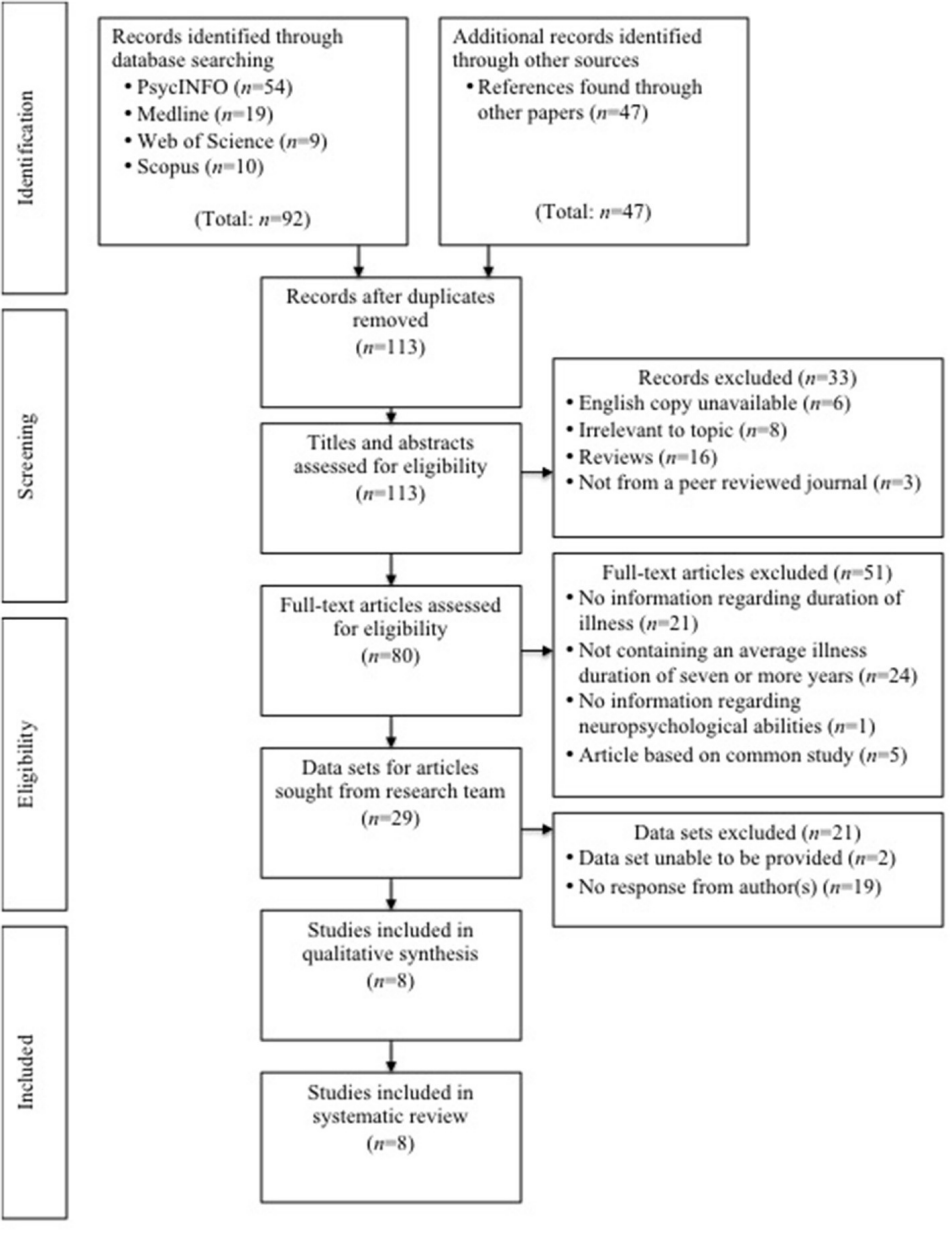
PRISMA flow diagram of study selection.

Key search terms included (anorexia nervosa) AND (severe OR enduring OR chronic OR persistent OR longstanding) AND (neuropsychology). The second author independently screened 10% of the titles and abstracts in order to control for selection bias, resulting in agreement over the selected articles. A flow diagram of the various stages of the selection process based on the PRISMA guidelines is presented in Figure 1.

### Selection Criteria

#### Inclusion Criteria

Studies were required to include the following:

a. Contain information regarding the condition in humans only (of either gender);
b. Include participants of any age group;
c. Publications which included information regarding duration of illness;
d. Contain information relating to AN that was severe and enduring, with the average illness duration required to be seven or more years; and
e. Contain information regarding neuropsychological abilities.

The current review included:

a. Study designs including but not limited to Randomised Control Trials (RCTs), with the exclusion of reviews;
b. Any setting;
c. All dates of publication for records outside of the database search, as well as all dates available when searching the databases of PsycINFO (1806 - present), Medline (1950 - present), Web of Science (1900 - present), and Scopus (1960 - present); and
d. The requirement that publications be peer-reviewed journal articles.

#### Exclusion Criteria

Studies were excluded if they:

a. Did not explicitly examine an AN population (i.e., articles exclusively examining other eating disorders such as Bulimia Nervosa);
b. Explored Anorexia in the context of a co-morbid condition to another illness (i.e., anorexia as opposed to AN);
c. Were a secondary publication of the same data;
d. Were unable or unwilling to provide the data set for the participants that were ill with AN for a 7 or more year period of time;
e. Did not report neuropsychological performance; or
f. Were published in a language other than English.

### Data Extraction

Data collected included the neuropsychological ability being assessed and the measure used for assessing that ability. Authors from eligible articles were contacted and requested to provide data only for participants within their study who had experienced an AN illness duration of 7 or more years. Individual data scores for these participants or descriptive statistics (means, standard deviations and sample sizes) for the main outcome measures in SE-AN participants were requested for the investigation of neuropsychological abilities. In addition, the following information was extracted from the article to aid the quality analysis: hypotheses, participant characteristics, participant response rate, power calculations, test setting, statistical measures, reliability and validity of outcome measures, main findings, random variability and potentially confounding variables.

### Quality Assessment

The methodological quality of the included studies were assessed using a modified version of the original Quality Index by Downs and Black (1998), with the original version designed for assessing studies relating to the quality of health care interventions. The modified version by Ferro and Speechley (2009) excludes some of the items from the original index that related specifically to interventions. This was deemed a more appropriate assessment based on the topic of the current systematic review.

The Ferro and Speechley (2009) quality index that was used to assess the quality of the research included in the systematic review consisted of 15 items and was scored dichotomously from 0 (no/unable to answer) to 1 (yes). It consisted of three subscales, which included reporting (0–7), external validity (0–3), and internal validity (0–4). The power of each study was assessed using a single item (0–1).

The primary author conducted the assessment with the second author independently assessing 10% of the articles in order to control for bias. Agreement was reached with articles representing higher methodological quality (≥10) resulting in no exclusion of articles.

### Data Analysis

The type of statistical techniques employed was determined by the data available for each analysis with no correction for multiple comparisons required given the independence of each test. Most data included in the current review suited an independent samples *t*-test to compare neuropsychological performance between the participants with a SE-AN presentation and a healthy control sample. In circumstances that allowed for statistically controlling a variable suspected of having an influence over the neuropsychological ability, an Analysis of Covariance (ANCOVA) was conducted in order to increase the power and sensitivity of the test. Confidence intervals [95%] were reported with small sample sizes warranting the use of Hedge's *g* when reporting the effect sizes for independent samples *t*-tests or partial eta squared for the ANCOVA analyses (Lakens, 2013). Effect sizes were evaluated in accordance with Cohen's *d* (Cohen, 1969) guidelines of a small (*d* = 0.2), moderate (*d* = 0.5), and large (*d* = 0.8) interpretation with the additional guidelines provided by Sawilowsky (2009) of a very small (*d* = 0.01), very large (*d* = 1.2) and “huge” (*d* = 2.0) interpretation. For the ANCOVA, effect sizes were evaluated using guidelines proposed by Cohen (1988) of a small ($\eta_{\text{p}}^{2}$ = 0.01), moderate ($\eta_{\text{p}}^{2}$ = 0.06), and large ($\eta_{\text{p}}^{2}$ = 0.14) interpretation. When a healthy control group was used in a study, this was adopted as the comparison group given this data was collected under the same testing conditions as SE-AN participants and was therefore less likely to bias the findings. In the context of no control group or a case study, the performance of participant(s) was compared to standardised norms. Assumptions for each test and the normality of data were assessed when individual data scores were provided and reported in the Results section after each analysis.

## Results

### Study Selection

The search retrieved a total of 139 records with 113 remaining after duplicates had been removed. Titles and abstracts were assessed, which resulted in 33 records being excluded for reasons including having no English copy available (*n* = 6), being irrelevant to the topic (*n* = 8), being a review (*n* = 16), or not being published in a peer reviewed journal (*n* = 3). The remaining 80 articles were assessed using the full-text, which resulted in 51 records being excluded for reasons including not containing information regarding duration of illness (*n* = 21), not containing an average illness duration of 7 or more years (*n* = 24), not containing information regarding neuropsychological abilities (*n* = 1), or the article being based on a common study (*n* = 5).

Authors from the remaining 28 articles (from the original search) were contacted on 18 November 2020 via email and were requested to provide data for only participants within their study that had experienced the AN illness for 7 or more years. For authors who were unable to be contacted directly, co-authors were included in the email trail with a request for data sets to be sent to the primary author of this review. In circumstances where there was no reply from authors within a 4-week period, a reminder email was sent on 15 December 2020, 3 days prior to the 1-month deadline set for authors to send the requested data sets. Extensions were offered in both emails sent to authors and granted in circumstances where the workload of authors or other reasons prevented them from delivering data sets by 18 December 2020. An updated search was conducted on 16 April 2021 with one additional article found to meet criteria. The primary author of this study was contacted via email on 16 April 2021 with the same request as described above.

Following the request from these collated searches, 21 records were excluded for being unable to provide the required data set (*n* = 2) or not receiving a response from the author(s) (*n* = 19). The remaining eight articles were subjected to a quality assessment with all being deemed eligible and therefore being included in the review (see Figure 1 for a flow diagram detailing the selection process based on the PRISMA guidelines; Moher et al., 2009).

### Descriptive Statistics

Data from a total of 166 participants with a SE-AN presentation was extracted from previously conducted research on eating disorders to explore the neuropsychological profile of this subpopulation. The neuropsychological abilities assessed through the eight studies included intelligence (*n* = 98), set-shifting (*n* = 51), central coherence (*n* = 9), decision making (*n* = 9), memory (*n* = 29), attention (*n* = 18), reasoning (*n* = 17) and information processing speed (*n* = 1). See Table 1 for study characteristics.

**Table 1:** Study characteristics.

**References**	**SEAN (*n*)**	**Age: Mean (*SD*; years)**	**Illness duration (years): Mean (*SD*)**	**BMI (kg/m^**2**^): Mean (*SD*)**	**Subtype (R/BP)**	**Neuropsychological Ability Measured**
Abbate-Daga et al. (2014)	41	30 (8) Range: 16–55	12.71 (6.40) Range: 7–30	15.13 (2.05)	R: 30 BP: 11	Set-shifting
Connan et al. (2006)	11	28.64 (6.83) Range: 20–42	11.33 (3.83) Range: 7–18	16.45 (2.21)	Data unavailable^a^	Intelligence Memory
Danner et al. (2012).	9	29 (4.44)	10.36 (2.46) Range: 7–11	14.35 (1.80)	R: 6 BP: 3	Set-shifting Central coherence Decision making
Goddard and Treasure (2013)	15	27.33 (5.26) Range: 20–36	11.67 (3.83) Range: 7–17	15.72 (1.84)	R: 10 BP: 5	Intelligence
Harrison et al. (2010)	23	32.39 (11.44)	15.39 (10.79)	15.23 (2.00)	R: 13 BP: 10	Intelligence
Hemmingsen et al. (2020)	1	35	25	8.41	R: 1	Memory Set-shifting Processing speed Attention
Schilder et al. (2016)	49	27.96 (8.15)	12.99 (7.66)	17.03 (2.11)	Data unavailable^b^	Intelligence
Seidel et al. (2021)	17	31.47 (10.32)	12 (4.17)	17.61 (2.04)	R: 13 BP: 4	Memory Attention Reasoning

a *Subtype data missing for seven participants*.

b *Subtype data not collected*.

Despite all studies being deemed eligible for inclusion according to the Ferro and Speechley (2009) quality index, data on potentially confounding variables were explored in each study to determine the degree of consideration by authors of their potential impact on neuropsychological performance. Based on previous research, it has been suggested that variables including severity of symptoms (Andrés-Perpiña et al., 2011; Bodell et al., 2014), comorbidity (Giel et al., 2012; Rose et al., 2012; Abbate-Daga et al., 2015), medication use (Herrera-Guzman et al., 2009; Krysta et al., 2015), Intelligence Quotient (IQ; Diaz-Asper et al., 2004; Hiscock, 2007), years of education (Ardila et al., 2010), and treatment (Rylander et al., 2020) have the potential to impact neuropsychological performance (see Table 2 for Potentially Confounding Variables reported in each study). Where possible, the current study controlled for potentially confounding variables during the analysis.

**Table 2:** Potentially confounding variables reported in studies.

**References**	**Clinical severity of symptoms**	**Comorbidity**	**Medication**	**IQ**	**Years of education**	**Treatment**
Abbate-Daga et al. (2014)	Yes	Yes	Yes	Yes	Yes	Yes
Connan et al. (2006)	Yes	Yes	Yes	Yes	No	Yes
Danner et al. (2012)	Yes	Yes	Yes	No	Yes	Yes
Goddard and Treasure (2013)	Yes	Yes	Yes	Yes	Yes	Yes
Harrison et al. (2010)	Yes	Yes	Yes	Yes	Yes	Yes
Hemmingsen et al. (2020)	Yes	Yes	Yes	Yes	Yes	Yes
Schilder et al. (2016)	Yes	Yes	No	Yes	No^a^	Yes
Seidel et al. (2021)	Yes	Yes	Yes	No^b^	Yes	Yes

*IQ, Intelligence quotient*.

a *Although discussed as associated with IQ, no information or provisions made for potentially confounding variable*.

b *Although discussed as associated with patient group, no information or provisions made for potentially confounding variable*.

### Intelligence

A highly debated concept (Flynn, 2007), intelligence has been a term used to describe mental capacity that is a product of both hereditary and environmental factors and includes the ability to reason, plan, problem solve, comprehend complex ideas, learn as well as think in an abstract manner (Gottfredson and Saklofske, 2009; Deary, 2013). Four studies explored intellectual functioning using three different measures. Connan et al. (2006) used a short form of the Wechsler Adult Intelligence Scale-Revised (WAIS-R; Wechsler, 1981) with a healthy control group used for comparison. An independent-samples *t*-test was conducted to compare the WAIS-R full scale as well as the verbal and performance subscales of this assessment between the severe and enduring proportion of the AN participants exclusively and the healthy control comparison group used in the study by Connan et al. (2006). There was no significant difference found between participants with a SE-AN presentation (*M* = 104.80, *SD* = 18.35) and healthy control participants (*M* = 115.91, *SD* = 16.77; *t* (19) = −1.45 (two-tailed), *p* = 0.16, mean difference between groups = −11.11, CI [−27.15, 4.93]) on the full-scale score^1^. The standardised mean difference between groups was moderate to large (Hedge's *g* = 0.61). Similarly, there were no significant differences found on the verbal and performance subscales between the two groups (for statistics on these subscales, see Table 3). All assumptions for this test were met.

**Table 3:** Between-group comparisons for WAIS-R subscales.

	**Mean (*SD*)**	**Mean (*SD*)**	***T*-test comparison**
	**SE-AN group (*n* = 10)^**a**^**	**HC group (*n* = 11)**	
Verbal subscale	108.60 (15.62)	115.36 (17.42)	*t* (19) = −0.93, *p* = 0.36 CI [−21.93, 8.41]
Performance subscale	100.30 (20.91)	112.55 (17.11)	*t* (19) = −1.48, *p* = 0.16, CI [−29.63, 5.13]

a *Data was missing on the WAIS-R scores in the original study for one participant with SE-AN*.

The National Adult Reading Test (NART; Nelson and Willison, 1991) was the measure of intelligence used by Harrison et al. (2010) as well as Goddard and Treasure (2013). The test involves participants reading aloud a list of words that are irregular in terms of their grapheme-phoneme correspondences, with scoring based on whether the individual pronounces the non-phonetic words correctly or incorrectly (Bright et al., 2002). A higher score on this measure indicates a greater number of correct pronunciations and therefore prediction of a higher IQ. This measure has been a commonly used research tool in predicting premorbid intelligence (Kiely et al., 2011; Norton et al., 2016). Using the Harrison et al. (2010) data on SE-AN participants exclusively, an independent samples *t*-test was conducted to compare this subpopulation with healthy control participants. There was no significant difference found between SE-AN (*M* = 112.65, *SD* = 9.92) and healthy control (*M* = 113.27, *SD* = 7.39) participants (*t* (111) = −0.33 (two-tailed), *p* = 0.74, mean difference between groups = −0.62, CI [−4.30, 3.06]) on the NART predicted IQ score. The standardised mean difference between groups was very small to small (Hedge's *g* = 0.08). With individual data scores provided for the SE-AN participants, normality was assessed and assumptions met for this group. However, with only descriptive statistics provided for the healthy control group as opposed to individual data points, assumptions of normality were unable to be tested for this comparison group.

An independent samples *t*-test was also conducted on the SE-AN participants exclusively as well as the healthy control group from a study by Goddard and Treasure (2013). A significant difference in the NART predicted IQ scores was found with the SE-AN group (*M* = 107.33, *SD* = 8.36) demonstrating superior performance compared to the healthy control group (*M* = 102.12, *SD* = 8.44; *t* (63) = 2.10 (two-tailed), *p* = 0.04, mean difference between groups = 5.21, CI [0.26, 10.17]). The standardised mean difference between groups was moderate to large (Hedge's *g* = 0.61) with the distribution of the data determined to be reasonably normal.

The Wechsler Adult Intelligence Scale, third edition (WAIS-III; Wechsler, 1997a) was the measure of intelligence adopted by Schilder et al. (2016). With no healthy control group recruited through the study, comparisons of IQ were made using the normative data provided by the WAIS-III (Administration and scoring manual; Technical manual; Wechsler, 1997a). Using an independent samples *t*-test on a single group, it was determined that there was no significant difference in scores between the SE-AN participants (*M* = 101.47; *SD* = 11.61) and the standardised normative data on the full-scale IQ score (*t* (48) = 0.89 (two-tailed), *p* = 0.38, mean difference between groups = 1.47, CI [−2.73, 5.67]). The standardised mean difference between groups was very small to small (Hedge's *g* = 0.10). Similarly, there were no significant differences found on the verbal and performance subscales between the two groups (for statistics on these subscales, see Table 4). Given descriptive statistics of the SE-AN sample were the only data provided by authors (not individual data scores), assumptions of normality were unable to be tested prior to the analysis for this group.

**Table 4:** Between-group comparisons for WAIS-R subscales.

	**Mean (*SD*)**	***T*-test comparison**
	**SE-AN group (*n* = 49)**	
Verbal subscale	102.65 (11.85)	*t* (48) = 1.57, *p* = 0.12 CI [−1.55, 6.85]
Performance subscale	100.61 (12.07)	*t* (48) = 0.35, *p* = 0.73 CI [−3.59, 4.81]

### Set-Shifting

As previously identified, set-shifting is regarded as one of the basic factors of executive functioning and relates to the ability to move back and forth effectively between different mental tasks (Ely et al., 2016; Lange et al., 2016). Set-shifting was investigated through three studies using the Wisconsin Card Sorting Test (WCST; Berg, 1948). Perseverative errors are the most commonly used indicator for determining set-shifting abilities on this test and accordingly was the outcome of interest in the current study (Li, 2004; Woicik et al., 2011; Westwood et al., 2016). An independent samples *t*-test was conducted to determine whether there was a significant difference in perseverative errors between the severe and enduring participants exclusively and the healthy control comparison group based on data from the Danner et al. (2012) study. This study used a computerised version (referred to in the study as the *Berg's Card Sorting Test*), which concluded after administering all 128 cards or completing nine categories. The analysis revealed no significant differences between participants with a severe and enduring presentation (*M* = 19.75, *SD* = 7.83) and the healthy control sample (*M* = 20.13, *SD* = 5.28; *t*(21) = −0.14 (two-tailed), *p* = 0.89, mean difference between groups = −0.38, CI [−6.07, 5.31]) with the standardised mean difference between groups very small to small (Hedge's *g* = 0.06)^2^. Given descriptive statistics was the only data provided by the authors (not individual data scores), assumptions of normality were unable to be tested prior to the analysis.

In the study by Abbate-Daga et al. (2014), a pen-paper version of the WCST was used with a total deck of 64 cards administered to participants. An independent samples *t*-test revealed a significant difference in perseverative errors between the sample containing the severe and enduring participants exclusively (*M* = 7.22, *SD* = 6.05) and the healthy control participants (*M* = 5.27, *SD* = 2.58; *t*(98) = 2.21 (two-tailed), *p* = 0.03, mean difference between groups = 1.95, CI [0.20, 3.70]). The standardised mean difference between groups was small to moderate (Hedge's *g* = 0.45). Given the absence of individual data scores, assumptions of normality were unable to be assessed prior to the independent samples *t*-test being conducted.

The final study explored the neuropsychological functioning of a single patient with a SE-AN presentation. Despite this case report by Hemmingsen et al. (2020) investigating set-shifting through five measures, the current study focused on the findings from the WCST (Berg, 1948) given its popularity and comparability to the other two studies in this review. With no healthy control sample, standardised norms were used as a comparison to determine set-shifting ability 2 weeks after the patient had been admitted into hospital for treatment^3^. It was determined that the patient was within the moderately impaired range based on the number of perseverative errors (1st percentile) compared to standardised norms.

### Central Coherence

Central coherence relates to the degree of cognitive bias in information processing (Lopez et al., 2008). One study investigated this function using the Rey-Osterreith Complex Figure Test (ROFT; Meyers and Meyers, 1995). Danner et al. (2012) adopted scoring procedures by Booth (2006), which involves two independent elements being measured and forming a coherence index, with higher scores indicating a more coherent drawing style (Lopez et al., 2008). An independent samples *t*-test was conducted with no significant difference identified on the central coherence index between participants with a severe and enduring presentation exclusively^4^ (*M* = 1.80, *SD* = 0.22) and a healthy control sample (*M* = 1.96, *SD* = 0.26; *t*(21) = −1.48 (two-tailed), *p* = 0.15, mean difference between groups = −0.16, CI [−0.39, 0.07]; see Table 5 for statistics on the copy and recall accuracy elements). The standardised mean difference between groups was moderate to large (Hedge's *g* = 0.62). Given descriptive statistics were the only data provided by authors (not individual data scores), assumptions of normality were unable to be tested prior to the analysis.

**Table 5:** Between-group comparisons for independent elements of the ROFT.

	**Mean (*SD*)**	**Mean (*SD*)**	***T*-test comparison**
	**SE-AN group (*n* = 9)**	**HC group (*n* = 15)**	
Copy accuracy	34.06 (2.51)	35.4 (0.83)	*t* (22) = −1.92, *p* = 0.07, CCI [−2.78, 0.11]
Recall accuracy	21.31 (6.24)^a^	25.1 (4.53)	*t* (21) = −1.68, *p* = 0.11, CI [−8.49, 0.91]

a *Data was missing on the WAIS-R recall accuracy scores in the original study for one participant with SE-AN*.

### Decision Making

Decision-making is a multi-dimensional process that has been hypothesised to relate to the functioning of the reward system. A series of motivational processes drive the individual to decide on a series of actions in order to achieve a desired outcome (Adoue et al., 2014; Chan et al., 2014). One study explored decision-making using the Iowa Gambling Task (IGT; Bechara et al., 1994). Danner et al. (2012) administered a computerised version of the original test with participants required to choose one card at a time from a deck of four cards across 100 trials. An advantage or disadvantage to the player occurs depending on the deck from which the card was selected with the IGT total score (advantageous choices minus disadvantageous choices) being the parameter most commonly of interest in systematic reviews (see Guillaume et al., 2015; Evens et al., 2016; Kovács et al., 2017). This parameter can range from −100 to +100 with positive scores indicating a higher selection of advantageous card selections and a negative score indicating riskier decision-making processes. In the current review, the IGT total score and the five blocks were investigated in order to assess the IGT performances over time (a similar method adopted by Rotge et al., 2017).

Independent samples *t*-tests were used to investigate decision making with a statistically significant difference in the IGT total scores found between the severe and enduring proportion of the AN participants (*M* = −10.22, *SD* = 21.94) and the healthy control participants (*M* = 15.07, *SD* = 20.66; *t* (22) = −2.84 (two-tailed), *p* = 0.01, mean difference between groups = −25.29, CI [−43.77, 6.81]). The standardised mean difference between groups was large to very large (Hedge's *g* = 1.16). In the between-group comparisons, a significant difference was also identified between SE-AN and healthy control participants in Blocks 2 and 3 (see Table 6 for descriptive statistics). Given descriptive statistics were the only data provided by authors (not individual data scores), assumptions of normality were unable to be tested prior to the analysis.

**Table 6:** Between-group comparisons for IGT blocks.

	**Mean (*SD*)**	**Mean (*SD*)**	***T*-test comparison**
	**SE-AN group (*n* = 9)**	**HC group (*n* = 15)**	
Block 1	−3.33 (6.32)	−3.07 (5.23)	*t*(22) = −0.11, *p* = 0.91, CI [−5.20, 4.68]
Block 2	−2.89 (4.01)	2.27 (4.13)	*t*(22)= −2.99, *p* = 0.01^*^, CI [−8.73, 1.59]
Block 3	−3.11 (8.01)	6.80 (7.63)	*t*(22) = −3.02, *p*=0.01^*^, CI [−16.70, −3.12]
Block 4	−2.00 (7.42)	5.47 (10.15)	*t*(22) = −1.92, *p* = 0.07, CI [−15.56, 0.62]
Block 5	1.11 (9.17)	3.6 (10.15)	*t*(22) = −0.60, *p* = 0.56, CI [−11.06, 6.08]

* *Statistically significant difference between groups*.

### Memory

Although many forms of memory exist, in a general sense this neuropsychological ability is regarded as the persistence of learning, which can later be revealed if encoded and stored effectively (Wechsler, 1997b; MacPherson et al., 2016). Memory was assessed in three studies with different measures being adopted. Connan et al. (2006) used the Doors and People Test (Baddeley et al., 1994), which has the purpose of comparing recognition and recall performance for both verbal and visual information (MacPherson et al., 2016). After initially conducting a one-way analysis of variance on the data by Connan et al. (2006), it was determined that an adjustment for the WAIS-R full scale IQ would be a more appropriate analysis with the relationship between the conditions and memory moderated by intelligence. A one-way between-groups ANCOVA was conducted with all preliminary checks confirming that there was no violation of assumptions for this test. After adjusting for intelligence, there was no significant difference in memory between the participants with a SE-AN presentation and healthy control participants, *F*_(1,17)_ = 0.40, *p* = 0.54, with a partial eta squared effect size that was small to moderate ($\eta_{\text{p}}^{2}$ = 0.02). It was determined there was a strong relationship between memory and intelligence ($\eta_{\text{p}}^{2}$ = 0.21; See Table 7 for descriptive statistics).

**Table 7:** Descriptive statistics for the measure of memory in the study by Connan et al. (2006).

	**SE-AN group (*n* = 9)^**a**^**	**HC group (*n* = 11)**
Unadjusted Mean (*SD*)	12.00 (3.08)	11.91 (4.32)
Adjusted Mean (SE)	12.51 (1.19)	11.49 (1.07)

a *Data from original study was missing on ‘Doors and People' test for two participants with SE-AN*.

The second study by Seidel et al. (2021) utilised the CogTrack^TM^ system, which is a program comprising of online cognitive tasks that assesses major aspects of cognitive functioning (Wesnes et al., 2017). Measures relating to memory that were assessed included the Quality of Memory score as well as the following three index scores: Working Memory Capacity Index (comprised of accuracy scores from the “Spatial Working Memory” and “Numeric Working Memory” tasks); Episodic Memory Capacity Index (comprised of accuracy scores from the “Word Recognition,” “Immediate Word Recall,” “Delayed Word Recall,” and the “Pattern Separation” tasks with consideration for the number of errors); and Speed of Retrieval Index (comprised of the reaction time scores for “Numeric Working Memory,” “Spatial Working Memory,” “Word Recognition,” and “Pattern Separation” tasks). Using independent samples *t*-tests, no measure relating to memory were found to be significantly different when comparing the severe and enduring proportion of the AN sample and the healthy control participants (see Table 8 for descriptive statistics). Given descriptive statistics were the only data provided by authors (not individual data scores), assumptions of normality were unable to be tested prior to the analysis.

**Table 8:** Between-group comparisons for measures of memory.

	**Mean (*SD*)**	**Mean (*SD*)**	***T*-test comparison**
	**SE-AN group (*n* = 17)**	**HC group (*n* = 36)**	
Quality of memory	358.92 (59.87)	364.55 (71.29)	*t*(51) = −0.28, *p* = 0.78, CI [−45.75, 34.49]
Working memory capacity index	91.22 (7.13)	92.37 (5.96)	*t*(51) = −0.62, *p* = 0.54, CI [−4.90, 2.60]
Episodic memory capacity index	149.47 (44.50)	152.42 (52.76)	*t*(51) = −0.20, *p* = 0.84, CI [−32.68, 26.78]
Speed of retrieval index	2535.85 (250.67)	2359.02 (360.74)	*t*(51) = 1.82, *p* = 0.07, CI [−18.24, 371.90]

The third study that included an assessment of memory performance was from a case report by Hemmingsen et al. (2020), which measured memory performance using the Wechsler Memory Scale III (WMS-III; Wechsler, 1997b). Using standardised norms as a comparison, the participant's scores indicate high average to very superior auditory, visual, immediate and general memory ability. Working memory performance indicates the patient's ability was within the average range (55th percentile).

### Attention

Attention is a cognitive process that involves focusing the mind in a clear and vivid manner on one task or object at the expense of others (Cohen, 2014). Two studies assessed attention with Seidel et al. (2021) using the CogTrack^TM^ internet-based system (Wesnes et al., 2017) and Hemmingsen et al. (2020) using the d2R Test of Attention – Revised (Brickenkamp et al., 2010). For the study by Seidel et al. (2021), three tasks created two composite scores for attention. The first was an Attentional Intensity Index (comprised of the sum of the reaction time scores for the “Simple Reaction Time,” “Digit Vigilance,” and “Choice Reaction Time” tasks), with an independent samples *t-*test revealing no significant differences between the severe and enduring proportion of the AN sample (*M* = 1234.33, *SD* = 156.98) and the healthy control participants (*M* = 1241.50, *SD* = 119.15; *t*(51) = −0.18 (two-tailed), *p* = 0.85, mean difference between groups = −7.17, CI [−85.27, 70.93]). The standardised mean difference between groups was very small to small (Hedge's *g* = 0.05). The second measure was the Sustained Attention Index (comprised of the sum of the accuracy scores for the “Simple Reaction Time,” “Digit Vigilance,” and “Choice Reaction Time” tasks), with an independent samples *t*-test revealing no significant differences between the severe and enduring proportion of the AN sample (*M* = 91.2, *SD* = 5.2) and the healthy control sample (*M* = 90.56, *SD* = 4.72, *t*(51) = 0.45 (two-tailed), *p* = 0.66, mean difference between groups = 0.64, CI [−2.24, 3.52]). The standardised mean difference between groups was very small to small (Hedge's *g* = 0.13). Given the absence of individual data scores, assumptions of normality were unable to be assessed prior to the independent samples *t*-tests being conducted.

The test utilised by Hemmingsen et al. (2020) provided an indication of selective attention whilst balancing the components of speed and accuracy. It is commonly scored by the number of processed targets whilst taking into consideration the number of errors (Hahm et al., 2020). Using standardised norms as a comparison, Hemmingsen et al. (2020) reported the participant to have made a number of processed targets within the low average range (21st percentile). Concentration performance was determined to be within the average range (42nd percentile) and the number of errors made within the high average range (>90th percentile).

### Reasoning

Reasoning is a multi-stage process whereby the combination of prior knowledge and new information allows for inferences to be drawn that will guide new thought patterns and/or human behaviour (Adolphs et al., 1996; Krawczyk, 2018). A study by Seidel et al. (2021) explored Grammatical Reasoning using the CogTrack^TM^ system (Wesnes et al., 2017), which was measured by an Overall Accuracy and Speed score. An independent samples *t*-test was conducted with no statistically significant difference in the Grammatical Reasoning Overall Accuracy between the severe and enduring proportion of the AN participants (*M* = 89.89, *SD* = 11.24) and the healthy control sample (*M* = 83.42, *SD* = 15.07; *t* (51) = 1.57 (two-tailed), *p* = 0.12, mean difference between groups = 6.47, CI [−1.79, 14.73]). The standardised mean difference between groups was small to moderate (Hedge's *g* = 0.46). The second measure was the Grammatical Reasoning Overall Speed with an independent samples *t*-test revealing no statistically significant difference between the severe and enduring proportion of the AN participants (*M* = 2739.42, *SD* = 491.75) and the healthy control participants (*M* = 2845.38, *SD* = 922.09; *t* (51) = −0.44 (two-tailed), *p* = 0.66, mean difference between groups = −105.96, CI [−585.69, 373.77]). The standardised mean difference between groups was very small to small (Hedge's *g* = 0.13). Given descriptive statistics were the only data provided by authors (not individual data scores), assumptions of normality were unable to be tested prior to the analyses.

### Information Processing Speed

The speed at which information is processed in the brain has been proposed as having a key function in facilitating higher order cognitive abilities such as executive functioning (Ising et al., 2014; Alloza et al., 2016). The case report by Hemmingsen et al. (2020) utilised the Processing Speed Index of the Wechsler Adult Intelligence Scale IV (WAIS-IV; Wechsler, 2008). Using standardised norms as a comparison, the participant performed within the average range (32nd percentile) on this index 2 weeks after admission into a hospital treatment program.

## Discussion

The aim of the current study was to synthesise available research into the neuropsychological functioning of SE-AN. To the author's knowledge, no systematic review has exclusively focused on the neuropsychological profile of this subgroup to date, allowing for only one prediction to have been made based on findings from the AN condition more generally. It was hypothesised that set-shifting would be impaired in individuals with a long-term experience based on evidence that this neuropsychological ability is hindered by a longer duration of AN (Lang et al., 2014; Talbot et al., 2015).

### Intelligence

Intelligence was explored through four studies, with one study (Goddard and Treasure, 2013) indicating a statistically significant difference in intellectual functioning with SE-AN participants performing superior to healthy controls. When interpreting these findings, there are three important issues to note. The first is that researchers need to consider the Flynn effect when measuring intelligence, which is the phenomenon of rising average IQ test scores observed across generations (Flynn, 2007). This has been proposed to be the result of increased health and nutrition, better education and improved standards of living in developed countries, with this effect having been observed across intelligence tests including pre-morbid estimates (Norton et al., 2016; Rindermann et al., 2017). With some of the studies in the current review using versions of tests that were standardised during earlier periods of time, such as Schilder et al. (2016) using the WAIS-III norms as a comparison group (which were originally published in 1997; Wechsler, 1997a), caution needs to be taken when interpreting these findings to ensure they have not been biased by this phenomenon.

The type of tests employed to measure intelligence also require attention given the symptomatology of AN. With low body weight a criterion for the illness, the effect of starvation on the brain is likely to have caused impairment during testing for SE-AN participants (see Bodell et al., 2014). It is important to consider whether measures of intelligence are appropriate or whether tests of pre-morbid intelligence provide a better measure of this ability in research on AN. Given the current review included both forms, it is debatable whether these findings are comparable.

A further consideration is the potential impact of years of education on intelligence measures (Ritchie and Tucker-Drob, 2018), which could result in an unfair disadvantage for participants with a long-term illness. Individuals with SE-AN are likely to have had schooling and tertiary education interrupted by hospital admissions and attendance at treatment facilities. Measuring intellectual ability as opposed to achievement levels can ensure AN participants are not disadvantaged, as well as considering the influence that schooling can have on measures of intelligence. In the current review, it was determined that two studies did not report years of education in participants (Connan et al., 2006 and Schilder et al., 2016), which requires caution when interpreting these findings. Given the conflicting evidence found in the current review, a greater exploration of the intellectual performance in individuals with SE-AN is warranted.

### Set-Shifting

Set-shifting has been consistently found to be negatively impacted by the AN illness (see Roberts et al., 2007; Tchanturia et al., 2011; Talbot et al., 2015) leading the authors to hypothesise that the severe and enduring participants would be significantly impaired on this ability compared to healthy controls. In the current review, three studies investigated set-shifting using the WCST (Berg, 1948) with the data analysis revealing mixed findings. The data set by Danner et al. (2012) revealed no significant differences between groups. In contrast, the severe and enduring participants demonstrated significantly more perseverative errors compared to healthy control participants in the data set by Abbate-Daga et al. (2014). This was a small to moderate effect with the case report by Hemmingsen et al. (2020) also revealing a participant with SE-AN to perform within the moderately impaired range in comparison to standardised norms. Although it is inappropriate to generalise the findings from a case study to the SE-AN population as a whole, the fact that there is mixed evidence across these three data sets warrants further investigation.

### Central Coherence

Like set-shifting, central coherence is a neuropsychological ability found commonly impaired in patients with AN (see Navon, 1977; Weinbach et al., 2017; Hamatani et al., 2018). Using only the SE-AN participants in the study by Danner et al. (2012), there was no evidence of a statistically significant difference in this ability compared to healthy control participants. However, with such a small sample size of SE-AN participants in this review (*n* = 9), the negative effect this would have had on the power of the analysis warrants further investigation.

### Decision-Making

Based on findings from the study by Danner et al. (2012), a significant difference in decision-making was observed with severe and enduring participants making poorer decisions compared to the healthy control comparison group. The magnitude of this difference was large and was found through the total score on the IGT as well as during two blocks of the test (Blocks 2 and 3; Bechara et al., 1994). Of interest is that the collective AN participants^5^ in the Danner et al. (2012) study did not perform significantly differently compared to healthy control participants on the IGT total score. It would appear that a significant difference only emerges when examining the severe and enduring participants within the AN sample, as opposed to the collective AN sample as a whole. This suggests that poor decision-making may be a neuropsychological variability in the profile of SE-AN, and potentially separate this subgroup from other stages of the AN illness. If additional research supports this finding, the identification and treatment of these individuals may be improved by testing for this neuropsychological trait during assessment. To the author's knowledge, this study was the first to provide evidence in support of the theory proposed by van Elburg et al. (2021) that poor decision-making skills are a characteristic of SE-AN. However, like central coherence, caution needs to be taken when interpreting these findings given the small sample size of SE-AN participants in this review (*n* = 9).

### Memory

There was no evidence of a significant difference in memory performance between individuals with SE-AN and healthy control participants using the three measures of memory included in this review. Of note was the strong relationship that emerged between memory and intelligence when re-analysing the data from the Connan et al. (2006) study. These findings suggest that a potentially confounding variable when assessing memory is intelligence and that future research should consider controlling for this variable to avoid biasing results. Like other abilities discussed in this review, the findings by Hemmingsen et al. (2020) on memory performance warrants caution in its interpretation given case reports may reflect individual differences as opposed to representing a unique characteristic of the SE-AN disease.

### Attention, Reasoning, and Information Processing Speed

In relation to attention and reasoning, the findings of the severe and enduring proportion of AN participants (as conducted in the current review) need to be considered in light of the findings relating to all AN participants (as conducted by Seidel et al., 2021). The non-significant findings in the current review are in contrast to the findings reported by Seidel et al. (2021) with the latter analysis indicating the Attentional Intensity Index as well as the Grammatical Reasoning Overall Accuracy measure were significantly different between AN participants and healthy controls. More specifically, AN participants demonstrated superior concentration and increased accuracy when reasoning compared to healthy control participants (Seidel et al., 2021), which was not found when using only the severe and enduring proportion of the sample.

The difference in findings from the study by Seidel et al. (2021) and the analysis from the current review may be explained by those with a more severe and enduring illness adapting to these impairments over time (with the original study containing individuals with a less chronic presentation, with illness duration *M* = 9.19, *SD* = 5.35). For the analysis conducted in the current study, the inclusion criteria required participants to have a minimum illness duration of 7 years, therefore comprising a more severe and enduring sample. Another explanation for the disparity in findings between the original and current examination are the differences in methods used for analysing the data. The study by Seidel et al. (2021) utilised an ANCOVA procedure, which allowed the authors to control for the variables of age, gender and education. The analysis conducted in the current review was unable to control for these variables as only descriptive statistics were provided. These findings may highlight the need to control for age, education and gender for future research investigating attention and reasoning abilities in this population.

As discussed, evidence from a case report requires further support before generalising to a subpopulation such as SE-AN. The case report by Hemmingsen et al. (2020) indicated the participant was performing within the low average range on attention and within the average range in respect to information processing speed. Although further research would be needed to confirm these findings, the results from the current study suggest that these three processes may not represent unique neuropsychological characteristics of SE-AN.

### General Discussion

The current review is an important step in determining whether there is a unique characteristic related to cognitive functioning that could assist in identifying patients with SE-AN. With duration of illness currently being the most frequently used criterion in identifying these presentations (Broomfield et al., 2017), investigating alternative characteristics is of paramount importance in order to continue research efforts into staging the disease (Maguire et al., 2008; Treasure et al., 2015).

As discussed, there is evidence to suggest that a longer duration of AN leads to more severe impairments in the functioning of the brain (see Dickson et al., 2008; Fonville et al., 2014; Lang et al., 2014). This is based upon the premise that neuropsychological deficits may develop as a result of a severe and enduring disease, given the long-term impact of starvation on the brain. Given that models such as the cognitive-interpersonal maintenance model of AN propose that cognition contributes to the development and maintenance of the illness (Schmidt and Treasure, 2006; Treasure and Schmidt, 2013), testing cognitive functioning in patients with SE-AN assists in proving or disproving these theories. If proven valid, it is only logical that an individual with a long-term experience of AN should present with a pronounced rigid cognitive processing style given their experience of maintaining the illness over a substantial period of time. To date, this has been the consensus (i.e., neuropsychological variability may develop over time, implying that cognitive functioning changes as a result, or rather, is caused by the long-term struggle with the illness). However, what if potential variations did not occur as a result of the disease, but alternatively, these variations caused the long-term trajectory of the AN illness? This latter hypothesis suggests that individuals may be born with or develop differences in their cognitive profile before developing AN thus making them more susceptible to experiencing a “chronic” form of the illness (like what occurs in SE-AN).

What evidence currently exists to support this alternative hypothesis is the study by Rose et al. (2016), which determined a unique neuropsychological profile to exist in a cluster of child and adolescent participants with AN. This particular sample ranged in age between 9 and 18 years (mean age of 15.67 years; Rose et al., 2016). However, whilst there is no information relating to the age range of individuals within the specific cluster of interest (which they referred to as “cluster 1”), it is unlikely that all participants could have met the most frequently used criterion in the literature for SE-AN, which is an experience of AN for a period of 7 or more years (Broomfield et al., 2017). If these individuals who consisted of 19% of the sample in the Rose et al. (2016) study do represent the ~20% of individuals that go on to develop SE-AN (Steinhausen, 2002), this is evidence to suggest that neuropsychological variability exists prior to the long-term experience of the AN illness. If this alternative hypothesis is valid, neuropsychological variability could be partially to blame for the long-term trajectory of the AN disease given it is a variable that differentiates other subgroups of AN patients from those who develop SE-AN.

Whether neuropsychological variability exists as a result of a long-term experience of AN or the alternative hypothesis that these differences pre-date the longevity of the illness, this research has the potential to provide crucial information in relation to the cognitive functioning of these patients. This is required when attempting to understand why current treatment options for AN have proven largely ineffective for patients with SE-AN (Dobrescu et al., 2020; Solmi et al., 2021). One of the most evidence-based supported treatment options for AN is Cognitive Behavioural Therapy (CBT; Dalle Grave et al., 2016), which has been adapted for eating disorders more generally (Fairburn, 2008; Fairburn et al., 2013; Dalle Grave et al., 2019). One of the principles of CBT is developing an awareness of alternative, more rational ways of thinking and responding to situations using adaptive behaviour. This is required when engaging in exercises including cognitive restructuring and when deciding on actions that may result in alternative outcomes in behavioural experiments (Murphy et al., 2010). Through the current review, it was revealed that decision-making was impaired in the sample of individuals experiencing SE-AN. Thus, the capacity to decide on alternative thinking patterns and choose adaptive behaviour may be compromised and hinder progress using a CBT approach (Johnco et al., 2013). Modifying first-line treatment options will need to be an area that is further explored if a unique neuropsychological profile is confirmed in future studies on SE-AN.

### Limitations

The current review was limited by having restricted access to individual data points, which prevented the testing of assumptions for some statistical analyses. Although some full data sets were provided (which allowed testing assumptions for independent *t*-tests and the ANCOVA), some independent *t*-tests needed to be performed using group means, standard deviations and sample sizes. This was an unavoidable limitation given ethical and privacy restrictions prevented some authors from providing full data sets, resulting in only descriptive statistics being allowed to be provided to the authors of the current review. Additionally, a limited number of studies in this area along with different measures being used to test neuropsychological processes prevented meta-analytic methods being conducted in the current review.

### Future Directions

First, empirical investigations dedicated to SE-AN is required with recruitment involving large enough sample sizes to allow power in confirming the findings of the current review. It is suggested by the authors that future research should use consistent tests when conducting neuropsychological research to reduce variability and allow for meta-analytic methods to be conducted. Second, consideration of confounding variables needs to be a priority when designing experiments. This is particularly important given the finding in the current review that there is a strong relationship between memory and intelligence. Third, researchers need to expand the comparison of SE-AN with other subgroups of AN. It was a requirement to first assess the neuropsychological functioning of SE-AN in comparison to a healthy control sample, which was achieved through the current review. The next phase would be to compare variability between stages of the illness, which will assist in confirming whether neuropsychological characteristics, such as poor decision-making, is an endophenotype of SE-AN. Following the discovery of the neuropsychological profile of SE-AN, it is recommended that efforts be made to determine whether any variability is pre-dated to the onset of the illness or instead the result of maintaining a long-term AN presentation.

## Conclusion

With research suggesting a long-term AN illness can change the structure and function of the brain (Treasure et al., 2015) and impact neuropsychological performance (see Dickson et al., 2008; Fonville et al., 2014; Lang et al., 2014), an investigation into the neuropsychological profile of SE-AN was warranted. The current review was an essential preliminary step in examining whether there were indications of SE-AN demonstrating neuropsychological variability. By drawing upon and extracting pre-existing data, the current systematic review was able to begin mapping the neuropsychological functioning of participants with a 7 or more year presentation of AN. Preliminary evidence from this study suggests that decision-making, intelligence and set-shifting may be features that represent variations in patients with SE-AN, which should be further examined. Future research confirming these findings and comparing this subgroup to other stages of the AN illness is required to continue mapping this profile. These research efforts will assist in determining whether early identification of these individuals is possible and improve approaches to treatment based on variations that may be found to pre-exist or develop as a result of this long-term disease.

## Data Availability Statement

The data analyzed in this study is subject to the following licenses/restrictions: The data is unable to be provided given the authors of this review do not own the rights to these data sets (data will need to be obtained from the original authors of these research studies).

## Author Contributions

CB was involved in conceptualising the idea for the review, conducting both the original and updated systematic searches, contacting authors to obtain data sets, analysing the data, conducting the quality analysis on articles, and writing the manuscript. KS was involved in conceptualising the idea for the review, independently screened 10% of the titles and abstracts during the systematic searches (to control for selection bias), independently conducting a quality assessment on 10% of the articles (to control for bias), and reviewed and edited the manuscript. ST was involved in conceptualising the idea for the review and provided supervision to CB as well as reviewed and edited the manuscript. All authors have seen and approved the manuscript being submitted.

## Conflict of Interest

The authors declare that the research was conducted in the absence of any commercial or financial relationships that could be construed as a potential conflict of interest.

## Publisher's Note

All claims expressed in this article are solely those of the authors and do not necessarily represent those of their affiliated organizations, or those of the publisher, the editors and the reviewers. Any product that may be evaluated in this article, or claim that may be made by its manufacturer, is not guaranteed or endorsed by the publisher.

## Footnotes

^1^Data was missing on WAIS-R scores in the original study for one participant with SE-AN.

^2^Data was missing in the original study by Danner et al. (2012) for one participant with SE-AN.

^3^Despite this patient being tested a total of three times, performance during the initial test was used in the current review to avoid bias that could have been caused by multiple exposures to the same test materials within a 12-month period.

^4^Data was missing on the CCI and Recall accuracy scores of the ROFT in the original study for one participant with SE-AN.

^5^Given there was an “AN currently ill” and “AN recovered” group in the Danner et al. (2012) study, this observation is only in reference to the former (“AN currently ill”) group.

## Abbreviations

AN: Anorexia nervosa
SE-AN: Severe and enduring anorexia nervosa.
